# Acknowledgment

**DOI:** 10.3402/gha.v6i0.20434

**Published:** 2013-02-25

The cluster of scientific papers ‘Public health in Vietnam: here's the data, where's the action?’ was published with the support of the Sida-funded project ‘Public Health Preparedness and Response to Critical Global Health Issues in Vietnam and Sweden’ which is being implemented by the Center for Health System Research, Hanoi Medical University, Hanoi, Vietnam, and Epidemiology and Global Health, Umeå University, Umeå, Sweden. The cluster was only possible because of the hard work carried out by the editors, mentors, and reviewers*:

**Table:** 

Ann Fitzmaurice	University of Aberdeen, UK
Fredrik Norström	Umeå University, Sweden
Heiko Becker	University of Heidelberg, Germany
Hans Stenlund	Umeå University, Sweden
Hoang Van Minh	Hanoi Medical University, Vietnam
Irina Campbell	NJ Child Health Project, USA
Jenni Hislop	Newcastle University, UK
Joacim Rocklöv	Umeå University, Sweden
Kim BaoGiang	Hanoi Medical University, Vietnam
Lars Lindholm	Umeå University, Sweden
Lars Weinehall	Umeå University, Sweden
Lena Wistrand	Co-Action Publishing, Sweden
Lennarth Nyström	Newcastle University, UK
Laura Ternent	Newcastle University, UK
Lucia D'Ambruoso	Umeå University, Sweden
Malin Eriksson	Umeå University, Sweden
Margareta Norberg	Umeå University, Sweden
Maria Nilsson	Umeå University, Sweden
Marie Lindkvist	Umeå University, Sweden
Nawi Ng	Umeå University, Sweden
Nguyen QuynhAnh	Hanoi School of Public Health, Vietnam
Nguyen Thanh Huong	Hanoi School of Public Health, Vietnam
Nguyen Van Huy	Hanoi Medical University, Vietnam
Pham NganGiang	Ministry of Health of Vietnam
Barbara Schumann	Umeå University, Sweden
Stig Wall	Umeå University, Sweden
Ta Thanh Van	Hanoi Medical University, Vietnam
Tran Thanh Huong	Hanoi Medical University, Vietnam
Tran Xuan Bach	Hanoi Medical University, Vietnam

*All editors, mentors, and reviewers involved in the peer review process had no competing interests or involvement with the papers to which they contributed as reviewers at the pre-submission stages.

